# Comparison of food and nutrient intakes between cohorts of the HAPIEE and Whitehall II studies

**DOI:** 10.1093/eurpub/ckv216

**Published:** 2015-12-04

**Authors:** Denes Stefler, Andrzej Pajak, Sofia Malyutina, Ruzena Kubinova, Martin Bobak, Eric J. Brunner

**Affiliations:** 1 Department of Epidemiology and Public Health, University College London, London, UK; 2 Department of Epidemiology and Population Sciences, Jagiellonian University, Krakow, Poland; 3 Institute of Internal and Preventive Medicine, Siberian Branch of the Russian Academy of Medical Sciences, Novosibirsk, Russia; 4 Novosibirsk State Medical University, Novosibirsk, Russia; 5 Centre for Health Monitoring, National Institute of Public Health, Prague, Czech Republic

## Abstract

**Background:** Differences in dietary habits have been suggested as an important reason for the large health gap between Eastern and Western European populations. Few studies have compared individual-level nutritional data directly between the two regions. This study addresses this hypothesis by comparing food, drink and nutrient intakes in four large population samples. **Methods:** Czech, Polish and Russian participants of the Health, Alcohol and Psychosocial Factors in Eastern Europe (HAPIEE) study, and British participants in the Whitehall II study, altogether 29 972 individuals aged 45–73 years, were surveyed in 2002–2005. Dietary data were collected by customised food frequency questionnaires. Reported food, drink and nutrient intake data were harmonised and compared between cohorts using multivariable adjusted quantile regression models. **Results:** Median fruit and vegetable intakes were lower in the pooled Eastern European sample, but not in all country cohorts, compared with British subjects. Median daily consumption of fruits were 275, 213, 130 and 256 g in the Czech, Polish, Russian and Whitehall II cohort, respectively. The respective median daily intakes of vegetables were 185, 197, 292 and 246 g. Median intakes of animal fat foods and saturated fat, total fat and cholesterol nutrients were significantly higher in the Czech, Polish and Russian cohorts compared with the British; for example, median daily intakes of saturated fatty acids were 31.3, 32.5, 29.2 and 25.4 g, respectively. **Conclusion:** Our findings suggest that there are important differences in dietary habits between and within Eastern and Western European populations which may have contributed to the health gap between the two regions.

## Introduction

High prevalence of unhealthy diets in Central and Easter Europe (CEE) and the former Soviet Union (FSU) has been suggested to play an important role in the high cardiovascular disease (CVD) mortality rates in these regions.^1–3^ Ecological data indicate that people in CEE and FSU consume less fruits and vegetable oils but more animal fats than individuals in Western Europe.^4^ However, comparison of individual-level dietary data between Eastern and Western European countries is rare.^5–7^

Nationally representative, individual-level nutritional surveys are conducted regularly in many European countries in order to monitor the population’s dietary habits. Although they provide good evidence for public health recommendations in the specific countries, their applicability for international comparison is limited because the dietary assessment methods differ between countries.^8^ Methods differ, to varying degrees, in terms of data collection tools, food classification, portion sizes and nutrient composition tables.^9–13^

The Health, Alcohol and Psychosocial Factors in Eastern Europe (HAPIEE) study is one of the largest and most recent studies with data on dietary habits of general population samples from the Czech Republic, Poland and the Russian Federation.^14^

In the current analysis, we compared individual-level food, drink and nutrient intakes between participants of the three HAPIEE cohorts and the UK-based Whitehall II cohort using identical methods for data analysis in both studies. Country-customised food frequency questionnaires (FFQ) with closely analogous design and layout were used for dietary data collection in the four cohorts.^15^^,^^16^

## Methods

### Study participants and dietary data collection

The design, recruitment process and dietary assessment of the HAPIEE and Whitehall II studies has been described previously.^14^^,^^17^

In brief, the HAPIEE study is a prospective cohort study, which is designed to investigate the relationship between traditional, non-conventional and psychosocial risk factors and chronic non-communicable diseases, particularly CVD, in CEE and FSU.^14^ The baseline survey in 2002–2005 recruited randomly selected population samples in Novosibirsk (Russia), Krakow (Poland) and six cities in the Czech Republic. Overall, 28 945 men and women (8857 Czechs, 10 728 Poles, 9360 Russians) aged 45–69 years at baseline were included in the study (overall response rate of 59%).

The Whitehall II study is a prospective cohort study of civil servants set up in 1985–1988 with the central aim to examine the impact of social inequalities on physical and mental health.^17^ Participants were recruited from 20 civil service departments in London; they undergo medical examination every 5 years and complete postal questionnaires between the screening phases. In the current analysis, we used dietary data from the seventh wave of the study which took place between 2002 and 2004, the same time as the baseline data collection of the HAPIEE study. In this phase, 6967 participants aged 50–73 years took part (68% of phase 1 responders).

In both studies, dietary data collection was carried out using a semi-quantitative FFQ. The FFQ used in the HAPIEE study was constructed on the basis of the Whitehall II study questionnaire. Participants could indicate how frequently they consumed a particular food or drink item using a 9-point scale ranging from ‘never, or less than once a month’ to ‘more than 6-times a day’.^15^^,^^16^

The FFQs completed by the Czech, Polish, Russian and UK cohorts consisted of 136, 147, 142 and 116 food and drink items, respectively. There were two reasons for the discrepancies: (1) Some food products were combined into one FFQ item in one country, but asked separately in others. For example, apricots, peaches and plums were combined in one question in the UK but in three separate questions in the HAPIEE cohorts. (2) Certain items were not included in all FFQs, because some of them were country-specific foods (e.g. pirogi, borscht). However, the majority of these FFQ-specific items (77, 66, 67 and 59% in the Czech, Polish, Russian and British questionnaires, respectively) were consumed in all four countries (e.g. pineapple, aubergine, cucumber, lasagne).

In all cohorts, participants who answered <90% of the FFQ questions and those who stated that the FFQ was not representative of their diet were excluded from the analysis. Participants with implausible food intake values, i.e. the bottom and top 1% of the cohort-specific energy intake/BMR ratio, were omitted.

Participants with missing data in any of the confounder variables were also excluded. Overall, 4473 British, 7298 Czech, 9098 Polish and 9103 Russian participants were included in the current analysis.

### Dietary data harmonisation

Measured intake of a given food group is likely to be proportional to the number of relevant items in the FFQ. Unless the differences between the FFQs represent country-specific differences in dietary habits (i.e. country-specific food items), which is not the case in the current comparison as described above, these discrepancies in the number of FFQ items may introduce reporting bias and need to be taken into account.

Firstly, we excluded those items from the analysis which were not common in all four FFQs. Secondly, regarding food and drink items which were asked separately in one but in combination in other FFQs, the portion/day intake levels were summarised and the data on the combined intakes were used in all cohorts. Overall, dietary intake data from 81 single or combined food and drink items were used in the current analysis.

Participants had to estimate their intake habits regarding an *average* portion or *medium-*sized food or drink item in all four FFQs. In order to calculate g day^−1^ intake of a specific item, standard portion sizes, provided by local dieticians, were used in previous analyses.^15^^,^^16^ These country-specific portion sizes were identical or similar for most items, however, for 29 (36%) of 81 items the difference was >50%. Although some of the small differences might reflect real regional differences, large discrepancies are likely due to arbitrary choices made by local dieticians during the construction of the FFQs. To avoid information bias due to different portion sizes, the g day^−1^ intake of each food and drink items was recalculated by substituting identical portion sizes in all cohort-specific datasets, using the portion sizes published by the UK’s Food Standard Agency.^18^ Alcoholic drink sizes were an exception, because the size of a standard drink clearly differs between countries and the questions on the FFQs were asked in line with the local habits. (i.e. 1 beer is 1/2 pint = 287 ml in the UK but 1 glass = 250 ml in CEE/FSU.)

In the HAPIEE cohorts, participants were asked to estimate their eating habits over the past 3 months. In contrast, the questions referred to the previous year in Whitehall II study, and regarding seasonal foods (i.e. fruits, vegetables), participants were asked to estimate their intakes in the time period when that particular item is in season. In order to eliminate the differences due to the different reference periods of the FFQs, we compared weighted intake data for fresh fruits and vegetables: for those participants of the HAPIEE cohorts who completed the FFQ during winter or spring, the intake of fresh fruits and vegetables were multiplied by the within-cohort summer–autumn vs. winter–spring ratio of median fresh fruit and vegetable intake.

National Food Composition tables and databases (FCDs) differ in completeness, accuracy and may use different analytical methods to measure nutrient content of foods. Because these technical differences in FCDs can lead to biased international comparisons of nutrient intake levels,^10^^,^^19^ we used the McCance and Widdowson’s FCD to estimate nutrient intake levels in both Whitehall II and HAPIEE cohorts.

### Further data preparation and statistical analysis

The food and drink items listed in the FFQs were categorised into food/drink groups and subgroups according to the European Food Safety Authority’s Foodex2 food classification system.^20^ The comparisons were carried out on absolute intake values for food/drink groups and subgroups, and on energy standardised intake values (calculated by the residual method) for nutrients.^21^

To take account of possible information bias, food/drink groups and nutrients were categorised as fully, partially or not comparable between cohorts, according to the contribution of the 81 identical items to their total intake. Food/drink groups and nutrients were considered fully comparable if >80% of intake was provided by common items in all cohorts. If the contribution was 60–80% in one or more of the cohorts, they were considered partially comparable. If the contribution was <60% of intake in one or more of the cohorts then the food, drink or nutrient was not considered comparable and results were not shown.

In the multivariable adjusted models, quantile regression method was used because of the non-normal distribution of food, drink and nutrient intake data. All comparisons were adjusted for age (continuous), sex, energy intake (kJ day^−1^, continuous), marital status (married/cohabiting; single/widowed/divorced), highest level of education (primary or less; O-level/vocational; A-level/secondary; BA/BSc or higher), employment status (employed; retired; not employed/not retired), alcohol intake (abstainers; moderate drinkers: <15 g day^−1^ for women, <30 g day^−1^ for men; heavy drinkers: ≥15 g day^−1^ for women, ≥30 g day^−1^ for men), smoking (non-; ex-; current smokers), vitamin supplement usage (regular users; irregular or not users), leisure time physical activity (high: >15 MET-hours day^−1^; moderate: 5–15 MET-hours day^−1^; low: <5 MET-hours day^−1^) and medical history (CVD or DM in medical history; no CVD or DM in medical history).

All statistical analyses were carried out using STATA 13.1 statistical software (StataCorp., College Station, TX, USA).

## Results

On average, ∼75% of total food/drink and energy intakes were captured by the 81 identical items in each cohort (tables 1 and 2). However, this proportion varied across food/drink groups, nutrients and cohorts. For example, on average, 2.2% of vegetable oil intake was provided by the common item in the Russian sample, while nearly all (96.1–100%) of the fresh meat intake came from identical items in all four cohorts (table 1).

**Table 1:** Comparison of the FFQs used in the British, Czech, Polish and Russian cohorts

Overall food and drink categories	Food and drink groups and subgroups (FoodEx2)	No. items in FFQ				No. items identical across the four FFQs	Mean percentage of food and drink intakes from the identical items^a^			
		UK	CZE	POL	RUS		UK	CZE	POL	RUS
Foods of animal origin	Meat and meat products	9	15	14	15	8	98.2	76.2	81.5	86.2
	Animal fresh meat/animal offals	5	6	6	7	5	100.0	96.2	98.9	98.9
	Processed meat products/sausages and comminuted meat	4	9	8	8	3	92.1	40.5	56.2	53.7
	Milk and dairy products	9	13	15	12	6	25.4	49.4	50.2	59.8
	Eggs and egg products	1	1	1	1	1	100.0	100.0	100.0	100.0
	Fish, seafood, amphibians, reptiles and invertebrates	5	5	7	7	3	75.6	37.0	54.2	36.3
Foods of plant origin	Grains and grain-based products	15	10	10	10	7	72.6	74.1	72.1	66.1
	Fruits and fruit products	11	23	22	23	11	100.0	86.7	85.4	86.8
	Fresh fruits	8	20	19	20	8	100.0	85.5	84.1	81.6
	Processed fruit products	3	3	3	3	3	100.0	100.0	100.0	100.0
	Vegetables and vegetable products	18	25	28	26	16	94.9	79.9	72.5	87.2
	Vegetables (all non-products)^b^	18	22	24	23	16	94.9	89.0	86.2	94.2
	Vegetable products	0	3	4	3	0	na.	0.0	0.0	0.0
	Legumes, nuts, oilseeds and spices	6	6	4	6	4	87.9	60.4	100.0	78.5
	Starchy roots or tubers and products	4	3	3	3	3	84.2	100.0	100.0	100.0
	Sugar, confectionery and water-based sweet desserts	3	4	5	4	3	100.0	94.5	96.3	98.1
Foods of mixed origin	Animal and vegetable fats and oils	5	7	9	7	3	38.7	60.4	58.3	32.7
	Animal fats and oils	1	4	4	4	1	100.0	78.9	86.5	95.2
	Vegetable fats and oils	2	2	2	2	1	8.3	31.9	23.8	2.2
	Fats and oils of mixed origin	2	1	3	1	1	11.8	100.0	48.7	100.0
	Seasoning, sauces and condiments	6	3	4	3	3	64.2	100.0	95.4	100.0
	Composite dishes	10	8	13	13	3	58.5	64.7	47.9	41.0
Drinks	Alcoholic beverages	5	5	5	5	5	100.0	100.0	100.0	100.0
	Water and water-based beverages	2	4	2	2	2	100.0	25.0	100.0	100.0
	Coffee, cocoa, tea and infusions	5	2	3	3	2	89.3	100.0	98.4	99.2
	Fruit and vegetable juices and nectars	2	2	2	2	1	80.1	65.8	66.2	88.7
Total		116	136	147	142	81^c^	80.4	68.3	79.1	78.6

^a^Values were calculated for each participant (in g day^−1^) as follows: Intake from the 81 identical FFQ items*100/Intake from all items in the original FFQs, for each food/drink group and overall.

^b^Including: brassica vegetables; bulb, stalk and stem vegetables; fruiting vegetables; leafy vegetables; legume greens, sprouts; non-starchy root and tuber vegetables; fungi; marine algae, aromatic herbs or flowers.

^c^Including nine which included more than one items each (combined items).

na.—not applicable.

**Table 2:** Mean percentage of nutrient and energy intake from the identical items compared with the original FFQs in the four cohorts^a^

Nutrients/energy	UK	CZE	POL	RUS
Total carbohydrate (g day^−1^)	76.4	76.7	75.8	74.7
Sugar (g day^−1^)	81.0	78.2	76.5	83.9
Protein (g day^−1^)	75.1	75.3	74.2	72.1
Total fat (g day^−1^)	73.4	70.9	69.5	63.3
Saturated fat (g day^−1^)	74.8	76.9	75.3	71.0
Polyunsaturated fat (g day^−1^)	65.5	65.2	64.9	60.7
Trans fat (g day^−1^)	57.2	76.9	78.0	79.3
Cholesterol (mg day^−1^)	83.7	84.2	81.6	77.1
Alcohol (g day^−1^)	100.0	100.0	100.0	100.0
Non-starch polysaccharides (g day^−1^)	78.6	79.0	73.5	76.8
Vitamin C (mg day^−1^)	86.8	80.1	72.3	66.8
Beta-carotene (μg day^−1^)	91.7	89.7	89.8	94.9
Total energy (kJ day^−1^)	76.7	75.0	73.4	70.4

^a^Values were calculated for each participant as follows.

Intake from the 81 identical FFQ items*100/Intake from all items in the original FFQs, for each nutrient and energy.

Table 3 shows the medians (IQR) g day^−1^ intakes of foods and drinks which were considered fully or partially comparable across cohorts. Multivariable adjusted cross-cohort comparisons, using the UK values as reference, are also shown. Average total and fresh fruit intake was significantly lower in Russian and Polish participants but higher in Czechs compared with the UK cohort. Russians had the lowest fresh fruit intakes, with average consumption less than half of any other cohort. In contrast, vegetable intake was significantly higher in Russians but lower in Poles and Czechs compared with the British sample. British participants reported higher consumption of starchy roots, alcohol, coffee, tea, legumes and fruit juices, but less meat products, sweets and animal fats than any of the Eastern European cohorts.

**Table 3:** Average intake of foods and drinks in the British, Czech, Polish, Russian cohorts and the pooled Eastern European sample

Food groups and subgroups (FoodEx2)	UK	CZE		POL		RUS		POOLED Czech, Polish and Russian sample	
	(n = 4 473)	(n = 7298)		(n = 9098)		(n = 9103)		(n = 25 499)	
	Median^a^ (IQR)	Median^a^ (IQR)	P-value^b^	Median^a^ (IQR)	P-value^b^	Median^a^ (IQR)	P-value^b^	Median^a^ (IQR)	P-value^b^
**Fully comparable foods and drinks**^c^									
Animal fresh meat/animal offals	74.2	76.8	<0.001	76.8	<0.001	117.2	<0.001	85.2	<0.001
	(47.6–102.0)	(47.6–111.6)		(60.0–103.2)		(68.4–154.8)		(58.6–120.0)	
Eggs	7.0	7.0	1.0	21.5	<0.001	21.5	<0.001	21.5	<0.001
	(3.5–21.5)	(7.0–21.5)		(7.0–21.5)		(7.0–21.5)		(7.0–21.5)	
Fruits and fruit products	256.1	275.0	<0.001	212.6	<0.001	130.0	<0.001	188.0	<0.001
	(158.8–382.2)	(152.4–477.3)		(124.4–346.6)		(70.1–219.7)		(102.7–335.9)	
Fresh fruits	231.8	256.0	<0.001	190.2	<0.001	91.4	<0.001	162.8	<0.001
	(137.7–350.0)	(138.2–451.4)		(114.6–325.1)		(43.1–180.0)		(78.0–308.1)	
Processed fruit products	16.5	14.7	<0.001	9.5	<0.001	21.5	<0.001	14.7	<0.001
	(7.0–32.0)	(7.7–25.2)		(2.5–20.0)		(7.7–48.5)		(7.0–31.7)	
Vegetables (all non-products)^d^	246.1	185.0	<0.001	197.3	<0.001	291.6	<0.001	235.9	<0.001
	(170.6–337.5)	(113.7–293.8)		(128.1–303.6)		(225.6–381.0)		(145.6–334.1)	
Starchy roots or tubers	98.3	86.8	<0.001	86.8	<0.001	86.8	<0.001	86.8	<0.001
	(75.3–152.6)	(75.3–101.2)		(75.3–141.1)		(73.8–146.2)		(75.3–138.3)	
Sugars, confectionery and water-based sweet dessert	8.1	8.8	<0.001	19.6	<0.001	31.1	<0.001	19.1	<0.001
	(3.5–24.9)	(3.5–21.5)		(7.0–35.1)		(15.6–42.9)		(7.0–36.6)	
Alcoholic beverages (portion day^−1^)	1.0	0.3	<0.001	0.1	<0.001	0.1	<0.001	0.1	<0.001
	(0.4–2.5)	(0.1–1.0)		(0.0–0.2)		(0.0–0.5)		(0.0–0.5)	
Coffee, cocoa, tea and infusions	869.0	581.7	<0.001	675.0	<0.001	561.0	<0.001	675.0	<0.001
	(503.0–1055.0)	(390.0–690.0)		(503.0–975.0)		(475.0–855.0)		(475.0–883.0)	
**Partially comparable foods and drinks**^e^									
All meat and meat products	90.1	92.2	<0.001	105.2	<0.001	135.5	<0.001	110.2	<0.001
	(59.8–122.7)	(59.8–130.9)		(80.0–136.4)		(92.2–179.3)		(76.3–151.5)	
Grains and grain based products	188.1	162.0	0.981	190.7	<0.001	218.5	0.002	190.5	<0.001
	(127.8–267.0)	(107.7–228.4)		(134.8–263.3)		(137.2–296.3)		(127.2–268.6)	
Legumes, nuts, oilseeds, spices	30.1	11.2	<0.001	11.2	<0.001	8.4	<0.001	11.2	<0.001
	(16.1–49.7)	(6.3–18.2)		(6.3–18.2)		(4.9–14.7)		(4.9–17.5)	
Animal fats and oils	0.0	1.4	<0.001	7.9	<0.001	4.3	<0.001	4.3	<0.001
	(0.0–4.3)	(0.7–10.0)		(0.0–25.0)		(1.4–10.0)		(0.7–10.0)	
Seasoning, sauces, condiments	10.8	12.2	<0.001	8.7	0.114	15.7	<0.001	12.2	<0.001
	(4.3–26.7)	(7.8–28.1)		(4.3–19.4)		(4.3–33.7)		(5.7–28.8)	
Fruit and vegetable juices and nectars	86.0	14.0	<0.001	28.0	<0.001	14.0	<0.001	14.0	<0.001
	(14.0–200.0)	(0.0–28.0)		(0.0–86.0)		(0.0–86.0)		(0.0–86.0)	

^a^Values are g day^−1^ intakes except for alcoholic beverages where portion/day intake is shown

^b^All P-values were calculated with quantile regression using the intake values in the UK cohort as reference category, adjusted for sex, age, energy intake, smoking, alcohol consumption, education, vitamin supplement intake, employment status, marital status, leisure time physical activity, CVD/diabetes in medical history.

^c^On average, more than 80% of their intake was provided by the common items (n = 81) in all four cohorts.

^d^Including: brassica vegetables; bulb, stalk and stem vegetables; fruiting vegetables; leafy vegetables; legume greens, sprouts; non-starchy root and tuber vegetables; fungi; marine algae, aromatic herbs or flowers.

^e^On average, 60–80% of their intake was provided by the common items (n = 81) in at least one of the cohorts, and more than 80% in the other cohorts.

Table 4 shows the medians (IQR) of energy-standardised nutrient intakes in the four cohorts, as well as the results of the quantile regression analysis. Only alcohol and beta-carotene intakes were fully comparable across cohorts. There was higher intake of beta-carotenes but lower intake of vitamin C in Russians compared with the other cohorts, in line with the high vegetable and low fruit intake in this sample. Total fat, saturated fat and cholesterol intake were significantly higher in all three Eastern European cohorts than in the British sample, consistent with the food intake data. Alcohol consumption of British participants was the highest of any cohort.

**Table 4:** Average intake of nutrients in the British, Czech, Polish, Russian cohorts and the pooled Eastern European sample

Nutrients	UK	CZE		POL		RUS		POOLED Czech, Polish and Russian sample	
	(n = 4 473)	(n = 7298)		(n = 9098)		(n = 9103)		(n = 25 499)	
	Median^a^ (IQR)	Median^a^ (IQR)	P-value^b^	Median^a^ (IQR)	P-value^b^	Median^a^ (IQR)	P-value^b^	Median^a^ (IQR)	P-value^b^
Fully comparable nutrients^c^									
Alcohol (g day^−1^)	10.9	2.6	<0.001	0.6	<0.001	1.1	<0.001	1.2	<0.001
	(3.4–28.4)	(0.6–9.8)		(0.0–2.4)		(0.0–4.8)		(0.0–4.9)	
Beta-carotene (mg day^−1^)	6.3	5.1	<0.001	7.3	<0.001	11.5	<0.001	7.8	<0.001
	(3.7–8.7)	(3.6–8.0)		(4.6–10.4)		(7.8–14.3)		(4.7–12.1)	
Partially comparable nutrients^d^									
Total carbohydrate (g day^−1^)	234.8	220.4	<0.001	225.6	<0.001	225.5	<0.001	224.4	<0.001
	(205.1–261.3)	(193.8–247.8)		(201.1–249.3)		(200.1–249.7)		(198.6–249.0)	
Sugar (g day^−1^)	116.1	108.3	<0.001	103.6	<0.001	107.4	<0.001	106.2	<0.001
	(94.4–139.1)	(83.3–136.9)		(83.5–127.2)		(86.9–129.0)		(84.8–130.4)	
Protein (g day^−1^)	72.3	78.4	<0.001	81.7	<0.001	82.0	<0.001	80.8	<0.001
	(63.9–81.7)	(68.3–88.1)		(73.3–90.7)		(71.4–93.0)		(71.1–90.8)	
Total fat (g day^−1^)	66.8	76.1	<0.001	78.0	<0.001	76.4	<0.001	76.8	<0.001
	(58.4–76.0)	(67.2–85.1)		(68.4–87.4)		(67.8–85.2)		(67.9–85.9)	
Saturated fat (g day^−1^)	25.4	31.3	<0.001	32.5	<0.001	29.2	<0.001	30.8	<0.001
	(21.3–30.1)	(26.9–36.2)		(27.1–38.7)		(25.0–33.7)		(26.2–36.1)	
Polyunsaturated fat (g day^−1^)	11.4	11.2	<0.001	10.7	<0.001	13.8	<0.001	11.7	0.715
	(9.5–14.2)	(9.6–13.2)		(9.0–12.7)		(10.9–17.5)		(9.7–14.4)	
Cholesterol (mg day^−1^)	218.3	308.9	<0.001	348.1	<0.001	320.0	<0.001	327.6	<0.001
	(172.2–272.3)	(255.7–371.0)		(295.2–403.8)		(263.5–387.2)		(272.0–389.3)	
Non-starch polysaccharides (g day^−1^)	16.6	15.8	<0.001	14.9	<0.001	14.4	<0.001	14.9	<0.001
	(14.0–19.8)	(12.6–19.9)		(12.4–18.0)		(12.4–16.7)		(12.4–18.0)	
Vitamin C (mg day^−1^)	143.6	136.5	0.003	109.3	<0.001	81.8	<0.001	105.5	<0.001
	(102.1–197.6)	(90.1–219.6)		(73.6–163.7)		(56.7–131.0)		(69.4–167.4)	
Total energy (MJ day^−1^)	7.4	6.4	<0.001	6.9	0.315	7.7	<0.001	7.0	0.892
	(6.1–8.9)	(5.1–8.1)		(5.6–8.3)		(6.2–9.5)		(5.6–8.7)	

^a^All values are energy standardised around 8 MJ day^−1^, except for alcohol and total energy intake for which absolute intakes are shown.

^b^All P-values were calculated with quantile regression using the intake values in the UK cohort as reference category, adjusted for sex, age, energy intake, smoking, alcohol consumption, education, vitamin supplement intake, employment status, marital status, leisure time physical activity, CVD/diabetes in medical history.

^c^On average, more than 80% of their intake was provided by the common items (n = 81) in all four cohorts.

^d^On average, 60–80% of their intake was provided by the common items (n = 81) in at least one of the cohorts, and >80% in the other cohorts.

An important difference between the Whitehall II and HAPIEE study participants was that the British cohort was based on civil service office workers, while large proportions of the Eastern European cohorts were engaged in physical occupations. In a sensitivity analysis restricting the comparisons to office workers the results were substantially similar (Supplementary tables S1 and S2). Further, the results of comparisons were similar to the main findings when the analysis was carried out separately in males or females (Supplementary tables S3, S4, S5 and S6).

## Discussion

### Main findings

In this study, using data collection based on the same FFQ methodology across four samples, dietary intakes in the HAPIEE and Whitehall II cohorts were fully comparable only for a subset of foods, drinks and nutrients. Median fruit and vegetable intakes were significantly lower in the pooled Eastern European sample than in the British cohort. Notably, we found large variation in average consumption of these foods between the Czech, Polish and Russian cohorts, such that vegetable rather than fruit consumption was important in the Russian diet while fruit was important in the Czech diet. Although the consumption of animal fats, including saturated fatty acids and cholesterol, was only partially comparable between cohorts, the figures suggest that intakes were significantly higher in Eastern European participants compared with the British.

### Strengths and limitations

Our study has a number of limitations which needs to be taken into account when interpreting the results. First, none of the included cohorts are fully representative of their respective national populations as a whole. The sampling frame included only urban inhabitants in the HAPIEE cohorts and London-based civil servants in the Whitehall II study. Second, there was a relatively low response rate in the Eastern European cohorts and some loss of baseline participants by Phase 7 of Whitehall II study which reduces the generalisability of our findings. A study in Poland recently found that hypertensive adults who live in rural areas consumed more fat and cholesterol but less carbohydrates and fibre than urban inhabitants.^22^ Particularly high-fat intake was also reported in a rural Lithuanian sample in the CINDI survey.^23^ This suggests that in the Polish sample, and probably in the other two Eastern European cohorts as well, the average intake of fats and other nutrients may have been higher if the HAPIEE cohorts had included rural participants. Individuals in non-manual occupations tend to have a better-quality diet than manual workers,^24^ indicating that participants of the Whitehall II cohort probably have healthier dietary patterns than the general UK population.

The FFQ is a cost-effective instrument to provide information on habitual diet in large studies. While the method has weaknesses of imprecision and information bias,^25^^,^^26^ the extent of random and systematic error stemming from these weaknesses is likely to be similar in all the cohorts we studied. Thus, the major impact on between-country comparisons was probably to reduce power to detect small differences in intake. Further, cross-cohort comparability of the dietary intake data was maximised since all FFQs used the same 9-point scale answer–options for all food and drink items, and strong emphasis was put on data harmonisation in the analytical phase. On the other hand, despite these efforts, many foods, drinks and nutrients were only partially comparable across cohorts. Regarding these, the interpretation of results is limited because a significant proportion of intake was unknown.

Further strengths of our study were the large sample sizes and contemporaneous data collections, between 2002 and 2005, in all four cohorts.

### Interpretation

Ecological data suggested that, on the aggregate level, fruit consumption is lower in CEE/FSU countries compared with Western Europe; however, there is probably no large difference in vegetable intake.^4^ Although this study confirms these previous findings, it also shows that important differences exist between countries *within* the Eastern European region. In Russia, the very low reported fruit intake is consistent with FAO data^4^ and it adds to the evidence that public health campaigns focusing on fruit consumption may be useful. On the other hand, high vegetable intake in this cohort is a favourable finding. To some extent it is probably due to widespread consumption of low-cost home-grown products. According to the Russian Statistical Office, 69% of vegetables produced in the country in 2012 came from household gardens, including dachas.^27^

The observation of significantly higher intakes of animal fat in the Eastern European cohorts compared with the British cohort confirms previous data and supports the hypothesis that its consumption plays an important role in the high CVD rates in these countries. Zatonski *et al.*^28^ suggested that substitution of animal fats with vegetable oils during the 1990s was one of the main reasons for the rapid decline in ischemic heart disease mortality rates in Poland. Although the comparability of fat intake, as well as the generalisability of our findings, is limited, the results indicate that the gap in animal fat intake between East and West still existed in the first half of the 2000s. This area of diet should probably be one of the central targets of the public health interventions in the Czech Republic, Poland and Russia.

Research suggests that intake of foods and drinks with high added sugar content are related to increased risk of obesity, diabetes and CVD.^29–31^ Although sugar intake (including all mono- and disaccharides) was the highest in British subjects, this result is probably due to the large contribution of fructose consumed via fruits and vegetables in this country cohort. The intakes of sweets and confectioneries were especially high in Poles and Russians. Added sugar consumption in Eastern European countries and its contribution to the high CVD rates would be worth examining in further studies.

## Conclusion

Despite the limited direct international comparability of many food groups and nutrients, our study supports hypotheses proposing that inadequate fruit and high animal fat consumption contributed to poor vascular and metabolic health status in several Eastern European countries in the early 2000s. The results indicate that there are important differences in dietary habits within CEE and FSU, such that dietary and nutritional recommendations are relevant across the whole region, but public health interventions need to be tailored to specific countries.

## Supplementary data

Key points

- Important differences in dietary habits exist between Eastern and Western European populations.
- This study found significantly lower fruit and vegetable intake but higher animal fat consumption in a pooled sample of Czech, Polish and Russian individuals compared with British civil servants. However, large variation in intake levels between the three Eastern European subsamples was also seen.
- The results support the hypothesis that unhealthy diet contributes to the high CVD rates of Eastern European populations.
